# Moderate Red Wine Consumption Increases the Expression of Longevity-Associated Genes in Controlled Human Populations and Extends Lifespan in *Drosophila melanogaster*

**DOI:** 10.3390/antiox10020301

**Published:** 2021-02-16

**Authors:** Juan Gambini, Lucia Gimeno-Mallench, Gloria Olaso-Gonzalez, Angela Mastaloudis, Maret G. Traber, Daniel Monleón, Consuelo Borrás, Jose Viña

**Affiliations:** 1 Freshage Research Group, Department of Physiology, Faculty of Medicine, University of Valencia, CIBERFES-ISCIII, INCLIVA, E46010 Valencia, Spain; juan.gambini@uv.es (J.G.); lucia.gimeno@uv.es (L.G.-M.); gloria.olaso@uv.es (G.O.-G.); jose.vina@uv.es (J.V.); 2 Linus Pauling Institute, Oregon State University, Corvallis, OR 97331-6512, USA; mastaloudis@gmail.com (A.M.); maret.traber@oregonstate.edu (M.G.T.); 3 Department of Pathology, Faculty of Medicine, University of Valencia, CIBERFES-ISCIII, INCLIVA, E46010 Valencia, Spain; daniel.monleon@uv.es

**Keywords:** lifespan, cardiovascular health, metabolic profile, resveratrol, phytoestrogens

## Abstract

The beneficial effects of moderate red wine consumption on cardiovascular health are well known. The composition of red wine includes several compounds, such as the phytoestrogen resveratrol, that exert these beneficial effects, although not all the mechanisms by which they act are known. Our aim was to study the effect of red wine consumption on longevity-related genes in controlled human populations, such as cloistered nuns. We found that the expression of catalase, manganese-superoxide dismutase, Sirt1, and p53 was increased in peripheral blood mononuclear cells after 14 days of moderate red wine consumption. This increase was accompanied by an enhanced metabolic wellness: fatty acids, cholesterol, branched chain amino acids (isoleucine and leucine), ketone bodies (acetoacetate), bacterial co-metabolites (trimethylamine), and cellular antioxidants (taurine) contributed to a change in metabolic profile after moderate red wine consumption by the nuns. No serious unwanted side effects were observed. Finally, we tested the effect of moderate red wine consumption on longevity in a controlled animal population, such as *D. melanogaster*, and found that it increased average life span by 7%. In conclusion, moderate red wine consumption increases the expression of key longevity-related genes and improves metabolic health in humans and increases longevity in flies.

## 1. Introduction

The beneficial effects of moderate red wine consumption were known from the times of Hippocrates [1]. This benefit was occasionally suggested in modern biochemical studies [2], but it was in 1992 when the French paradox was proposed [3]. This was a landmark study that showed that in spite of high consumption of saturated fats, the French population had low indices of cardiovascular risk [3,4] when compared with other countries with otherwise similar dietary habits. The difference was attributed to the high consumption of red wine in France.

Health benefits of moderate red wine consumption have been studied during the past decades, first in observational studies, and more recently, in experimental settings and randomized controlled studies. Cardiovascular beneficial effects were observed in humans after ingesting 400 mL of wine daily for four weeks as there was a significant increase in their high density lipoprotein (HDL) level; however, this did not occur when white wine was used [5]. Especially interesting is the study published by Hung et al. which showed that moderate alcohol consumption was associated with slower HDL-cholesterol decreases (followed for 6 years) compared with never drinkers, past, light, and heavy drinkers. Moreover, they found that the effect was different with the different ethanol-containing beverages studied [6]. A lowering of low density lipoprotein (LDL) oxidation and platelet aggregation associated with red wine ingestion has been reported in mice [7] and in humans [8]. Indeed, it has been very recently published that red wine drinking, consumption with food, and spreading alcohol intake over 3–4 days, were associated with a lower risk of mortality and vascular events among regular alcohol drinkers, after adjusting for the effects of average amount consumed [9]. Moreover, moderate red wine consumption exerts beneficial effects on endothelial dysfunction, dyslipidemia, hypertension, or metabolic diseases because of its antioxidant [10,11] or anti-inflammatory properties [12,13]. In the Prevention with Mediterranean Diet (PREDIMED) study, the intake of red wine was associated with improvements in four out of five criteria for metabolic syndrome (MetS criteria) (i.e., elevated abdominal obesity, low HDL-c levels, high blood pressure, and high fasting plasma glucose levels) [14]. A very interesting systematic review and meta-analysis of the impact of red wine polyphenols on vascular health has been recently published by Samuel R. Weaver et al. concluding that red wine and their components have the potential to improve vascular health in at risk human populations, particularly in regard to lowering systolic blood pressure, although such benefits are not as clear as those observed in animal models [15]. These studies could explain, at least in part, the French paradox.

The major aim of our study was to determine the effects of moderate red wine consumption on longevity and longevity-associated genes in human and *Drosophila* populations under controlled conditions. For this purpose, in this study we have used gene expression analysis in mononuclear cells to study the effect of moderate wine consumption on the expression of genes related to longevity in controlled populations of humans. The major shortcoming for nutritional intervention studies, especially those on nutrients like wine, is lifestyle. It is likely that individuals with moderate wine consumption also engage in other healthy habits, including stress control. Probably, the only populations that can be strictly controlled are monks and nuns who live in the same environment, with the same routines, and with controlled diets. We thus determined whether administration of red wine increases longevity-associated genes in humans. We cannot test the effect of wine on longevity itself in humans, and for this purpose, we used *D. melanogaster*, a well-established longevity model, useful for mapping genetic and nutritional interventions. Our results show that moderate red wine consumption increases the expression of longevity-associated genes in *D. melanogaster* and in humans and longevity in *D. melanogaster*.

## 2. Materials and Methods

### 2.1. Subjects of Study

We recruited 9 nuns (women aged 32–57 years) from two cloistered convents in Spain. The first study was performed on 4 nuns and the second one on 5, belonging to two different cloistered convents in the Valencian Community. All participants provided signed informed consent. A blood test and a medical examination was carried out before starting the test. The inclusion criteria were cloistered nuns who had not drunk wine or any other alcoholic beverage for at least the last two years and who wished to participate in the experiment. The exclusion criteria required that the nuns were not suffering from liver diseases or gastrointestinal problems or taking drugs whose pharmacodynamics could be affected by alcohol. All subjects gave their informed consent for inclusion before they participated in the study. The study was conducted in accordance with the Declaration of Helsinki, and the protocol was approved by the Ethics Committee of the Catholic University of Valencia. 

### 2.2. Nun Study Design

Blood and urine were obtained from each participant after an overnight fast, at the start of the study. We isolated peripheral mononuclear cells from blood on site. The samples were then frozen at −80 °C for later analysis. The trial lasted 14 days, during which all nuns consumed two glasses of red wine (200 mL each one) per day, one at lunch and one at dinner. A second blood test was performed after 14 days and proceeded in the same manner as in the first extraction. The nun’s food intakes during the intervention were registered by using Food Diaries filled in by the participants (n = 6). Energy provided by their diet was evaluated by using a specific dietetics software (DIAL v.3.11^®^ software (Alce Ingeniería^®^)). The percentage of energy provided by the diet relative to the required was 115% ± 16%.

### 2.3. Isolation of Mononuclear Cells

Blood samples were collected in Vacutainer^®^ Tubes containing Ficoll and then centrifuged in situ at 1500 g for 15 min. The supernatant (plasma) was transferred into sterile 2 mL Eppendorf tubes. The mononuclear cells present at the plasma–Ficoll interface were also collected in sterile 2 mL Eppendorf tubes and centrifuged at 1500 g for 5 min. Then the supernatant was removed, and the pellet was frozen with dry ice in situ.

### 2.4. Reverse Transcription

We used 1000 ng of total RNA preparation for reverse transcription. cDNA synthesis was performed using the “High-Capacity RNA-to-cDNA Kit” (Applied Biosystems, Thermo Fisher, Waltham, MA, USA), and a combination of random primers and oligo(dT) mix using the supplier’s protocols. Then, the obtained cDNA was diluted 1/10 in water and 1 μL of this dilution along with a synthetic competitor that had a single nucleotide polymorphism (SNP) mismatch served as templates for the competitive PCR that occurs in the MassARRAY Quantitative Gene Expression (QGE) protocol. PCR products were purified with shrimp alkaline phosphatase to remove excess of deoxynucleotide triphosphates (dNTPs). Then they were used for an extension reaction with a cocktail of extension primers and ‘ACT’ termination mix, containing ThermoSequenaseTM, dideoxynucleotide triphosphates ddATP, ddCTP and ddTTP, and the deoxynucleotide triphosphate dGTP as included in the kit ‘iPLEX GOLD’ (Sequenom Labcorp, San Diego, CA, USA) according to the supplier protocol. The final extension products were treated with SpectroCLEAN resin (Sequenom Labcorp, San Diego, CA, USA) to remove salts present in the reaction buffer, and 25 nL of reaction solution was dispensed onto a 384-format SpectroCHIPTM, where RNA quantification was undertaken. This analysis allows the estimation of the proportion of extension products corresponding to each different gene. Therefore, the absolute molecule number was calculated for each of the PCR templates. All peaks were ‘called’ and analyzed by MassARRAY Quantitative Gene Expression 3.4 software (Sequenom Labcorp, San Diego, CA, USA).

### 2.5. Quantification by MassARRAY Quantitative Gene Expression (QGE) Analysis 

**Figure 1:**
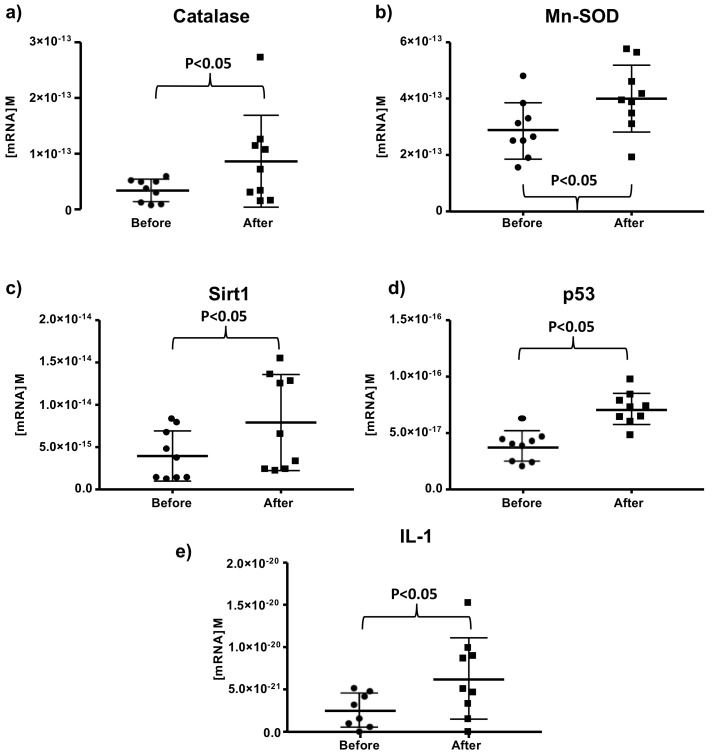
Moderate consumption of red wine increases catalase (**a**) MnSOD, (**b**), Sirt1, (**c**) p53, (**d**) and IL-1, (**e**) mRNA concentration in mononuclear cells. The concentration was measured by “sequenom quantitative gene expression (QGE) analysis”. Data are expressed as median and range with 95% CI for 9 different people, before starting the treatment and two weeks after a daily consumption of two glasses of red wine (200 mL, each), one at lunch and the other one at dinner.

Multiplexed primer and competitive template designs were created using the MassARRAY QGE Assay Design software v1.0 (Sequenom, Labcorp, San Diego, CA, USA) for random hexamer priming, such that at least one PCR primer spanned an exonic boundary per each transcript assayed. The gene panel assayed for this study was designed as a single multiplex reaction and the specific genes are shown in Figure 1.

Copy number determination for each transcript was conducted using real-time competitive PCR coupled with product resolution via Matrix-Assisted Laser Desorption and Ionization Mass Spectrometry (MassARRAY QGE, Sequenom, Labcorp, San Diego, CA, USA), as previously described [16]. Products were resolved on a linear MALDI-TOF mass spectrometer (MassARRAY Compact, Sequenom, Labcorp, San Diego, CA, USA). Signal acquisition, allele assignment, and peak area integration per spectrum were conducted with the MassARRAY retrotranscription (RT) Workstation v3.4 (Sequenom, Labcorp, San Diego, CA, USA). Data was analyzed using MassARRAY QGE Analyzer v3.4 (Sequenom, Labcorp, San Diego, CA, USA) with copy numbers for each transcript per sample determined based on the EC50 of standard curve titrations of known competitor amounts per assay vs. a fixed amount of cDNA template.

Normalization of copy numbers between samples for the different assays was conducted using a multiplexed set of 3 well-characterized human housekeeping (normalization) transcripts (beta-actin: ACTB-6, glyceraldenyde-6-phosphate dehydrogenase: GAPDH-6, and hydroxymethylbilane synthase: HMBS-7) and geNorm software. Normalization factors per sample were calculated using the geometric mean of the most stable combination of these normalization assays, determined by the measure of their pairwise variation as calculated by geNorm [17].

### 2.6. Determination of Plasma Biomarkers

Plasma α- and gamma-tocopherol concentrations were determined by high-performance liquid chromatography with electrochemical detection (HPLC-ECD), as previously described [18]. 

### 2.7. Determination of Urinary Biomarkers

Urinary F2-Isoprostanes (8-iso-15(R)-PGF2α, 8-iso-PGF2α, PGF2α, measured individually and subsequently summed) and urinary F2-Isoprostane metabolites (2,3-dinor-F1, 2,3-dinor-F2) were measured as described in detail in Taylor et al. [19], Using a single solid phase extraction cartridge, separation by HPLC, and detection by negative mode selected reaction monitoring mass spectrometry. 

Extraction and measurement of vitamin E urinary metabolites, alpha- and gamma-carboxyethyl hydroxychromanols (α-CEHC and γ-CEHC, respectively) were performed using a modified liquid chromatography–mass spectrometry (LC–MS) method of Leonard and Traber [20]. Urinary creatinine was measured using a kit from Sigma–Aldrich (St. Louis, MO, USA; kit#55A), which is based on the Jaffe reaction [21]. Urinary analytes are expressed per gram creatinine.

### 2.8. Storage, Preparation, and 1H NMR Spectroscopic Analysis of Blood Plasma for Metabolomics

We performed metabolomics using Nuclear magnetic resonance (NMR) spectroscopy. Blood plasma was stored at −80 °C and thawed before use. For NMR analysis, 400 μL of plasma was mixed with 200 μL of D_2_O phosphate buffer (as a field lock). A total of 500 μL of the mixture of each sample was then transferred into a 5-mm high-quality NMR tube individually. This procedure was performed by an automatic sample handler Gilson 215 (Gilson Inc, Middleton, WI, USA) and took no more than 2 min per sample. Thawed samples were kept at 6 °C in a SampleJet (Bruker GmbH, Rheinstetten, Germany) sampler exchanger before measurement and never more than 2 h. 1H-NMR spectra were recorded in a Bruker Avance DRX 600 spectrometer (Bruker GmbH, Rheinstetten, Germany) calibrated using standard reference samples of Alanine (10 mM) and Creatine (10 mM). Field homogeneity was tuned by semiautomated shimming for each sample following the instructions of the manufacturer. Samples were measured at 37 °C. All 1H NMR spectra were acquired using a standard one-dimensional pulse sequence with water suppression. A total of 256 instances of free induction decay (FID) were collected into 64 k data points with a spectral width of 14 ppm and the recycle delay (RD) of 1 s. The water signal was saturated with a weak irradiation during the recycling delay. Before Fourier transformation, the free induction decay was multiplied by a 0.3 Hz exponential line broadening. Spectral chemical shift referencing on the Alanine CH3 doublet signal at 1.475 ppm was performed in all spectra. We normalized each spectrum to the total aliphatic (0.5 to 4.2 ppm) spectral area. We used available spectral databases and 2D NMR experiments to aid structural identification of relevant metabolites. All spectra were processed using MNova (MestreLab) and transferred to MATLAB (MathWorks, Inc., Natick, MA, USA, 2006) using in-house scripts for data analysis. NMR metabolic signals were integrated and quantified using semi-automated in-house MATLAB (The Mathworks Inc., 2006) spectral analysis routines. The levels of metabolites were expressed as total relative metabolic content (calculated as the integral of the selected metabolite divided by the sum of all integrals in the spectra). The statistical significance of the differences at the 0.05 level was tested using the Anova two-tailed test with Bonferroni correction for multiple testing.

### 2.9. Multivariate Analysis of Metabolomic NMR Spectra

In multivariate data, the representation of any two variables against each other is not sufficient to obtain global correlations. PCA is a method for low-dimensional representation of multivariate data, in such a way that it optimally preserves the structure of the data. The PCA technique transforms variables in a data set into a smaller number of new latent variables called principal components (PC), which are uncorrelated to each other and account for decreasing proportions of the total variance of the original variables. Based on the assumption that larger variances represent more information, each new PC is a linear combination of the original variation such that a compact description of the variation within the data set is generated. Samples are assigned scores according to the variation measured by the PC with those having similar scores clustering together. The scores plot of the PCs allows the observation of most variations in the data set using a low number of variables and the detection of internal relationships within the data. The loadings of the different PCs provide information on which variables contain more information. Spectral regions between 0.5 and 4.5 ppm and between 5.5 and 9.5 ppm were binned in segments of 0.01 ppm width (6 Hz) and mean centered for multivariate analysis. The ethylenediaminetetraacetic acid (EDTA) regions (2.53–2.58, 3.1–3.3, and 3.6–3.7 ppm) and the ethanol regions (1.15–1.18 and 3.62–3.67 ppm) were excluded from the bucketing and multivariate analysis. We used Partial Least Squares (PLS)_Toolbox 6.7 (Eigenvector Research) for MATLAB to build the PCA models. 

### 2.10. Life Span in Drosophila melanogaster

Life span studies were conducted as described previously [22]. Flies tested in mortality experiments were reared at a constant density and were collected 1 ± 1 day post-hatching and maintained at 25 ± 1 °C and 50% of humidity. We collected 500 flies for each group (control and red wine). At least two replicate experiments were performed ∼300 flies/experiment. Flies (25/vial) were transferred to fresh vials containing standard medium (yeast-cornmeal-sugar-agar), and survivorship was scored every second day after collection. Control was composed of flies living in the ordinary conditions with standard diet of our *Drosophila* center, and the second, was composed of flies that were living in exactly the same conditions as controls except that red wine (10% *v/v*) was added to their diet.

### 2.11. Food Intake Determination in Drosophila melanogaster

We used the erioglaucine method. This reagent is added to the food, it stains the flies blue, and the intake can be estimated based on the amount of color. Erioglaucine blue 0.5% (*w/v*) was added to the food of all groups. The experiment started with one-week-old flies and was left in the food for 24 h. The flies were anesthetized on ice and homogenized with phosphate buffered saline (PBS) buffer in the following ratio: 20 flies for every 500 µL of PBS, subsequently the homogenate was centrifuged at 20,000 G for 10 min at 4 °C, 300 µL of the supernatant was poured collected, 400 µL of PBS was added to dilute it and the absorbance was measured at 625 nanometers (corresponding to the absorption maximum for erioglautia blue). The results were corrected taking into account the mg of protein in the fly homogenate.

### 2.12. RNA Extraction and the Real-Time RT Polymerase Chain Reaction (RT-PCR) in Drosophila melanogaster

Total RNA was isolated from flies using the QuickPrep Total RNA extraction kit (Amersham Pharmacia Biotech, Little Chalfont, UK). Each sample for RNA extraction was a pool of three flies. Reverse transcription and polymerase chain reaction were performed in one step using the TTh DNA polymerase kit (Roche Diagnostics, Risch-Rotkreuz, Switzerland). The mRNA expression was studied by real-time PCR (iCycler iQ real-time PCR detection system) using specific oligonucleotides for catalase, Cu/Zn superoxide dismutase, cytochrome c oxidase, Mn-superoxide dismutase, and 16S rRNA. The mRNA detection was carried out by measuring the binding of the fluorescent dye SYBR Green I to double-stranded DNA. The 18S RNA expression was used as housekeeping control. The threshold cycle (CT) was determined, and relative gene expression levels subsequently were calculated as follows: fold change = 2^−Δ(ΔCT)^, where ΔCT = CTtarget − CThousekeeping, and Δ(ΔCT) = ΔCTtreated − ΔCTcontrol.

### 2.13. Statistical Analysis

Data were represented by median and range with 95% CI. Normality of distribution was checked with the Shapiro–Wilk test, and homogeneity of variance was tested by Levene’s statistics. Comparison between groups was performed with a one-way ANOVA and two-tailed-test. Values <0.05 were considered statistically significant. The statistical significance of survival curves was assessed by the long rank test using IBM SPSS 21 software (IBM Software, Armonk, NY, USA).

## 3. Results

### 3.1. Effects of Moderate Red Wine Consumption on mRNA Levels of Longevity-Related Genes in Human Blood Mononuclear Cells

To investigate the effects of moderate red wine consumption on the expression of longevity-related genes, classified into different pathways related to ageing, we designed a Sequenom chip with 12 genes related to ageing (see Graphical abstract). The expression was measured in mononuclear cells before starting the treatment, and 14 days after a daily consumption of two glasses of wine per day (200 mL, each), one at lunch and another at dinner.

The result of Sequenom chip shows that two antioxidant genes, catalase and superoxide dismutase (Mn-SOD), were upregulated after red wine consumption for 14 days of (see Figure 1a,b). 

Moreover, we also found that red wine consumption resulted in an increase in the expression of important genes involved in ageing like p53 or sirtuin 1. Figure 1c,d shows that Sirt1 and p53 expression were increased in all nuns after wine consumption. So, we concluded there is a clear-cut activation in the expression of both p53 and sirtuin 1 when the nuns drank 200 mL of wine daily.

Moreover, due to the important role that the immune system plays in aging processes, we also determined the values of Interleukin 1 (IL-1) and we found that it was up-regulated only in some of the nuns, and the results were statistically significant. A total of 30% of the nuns did not show an increase in the expression of interleukin 1 (See Figure 1e). 

Of course, other longevity-associated genes were determined, and their expression did not change. Relevant among those are FOXO3, AKT1, sestrins, and telomerase (see Table S1).

### 3.2. Effect of Red Wine Consumption on Markers of Oxidative Stress and Levels of Antioxidant Vitamins

We set out to determine a number of parameters associated with vitamin levels or oxidative damage, especially to lipids. We did not find that wine consumption affected either antioxidant vitamins like vitamin E or markers of oxidative stress to lipids like isoprostanes (see Table S2).

Therefore, the beneficial effects of wine consumption are not directly related to protection from basal oxidative stress, but rather to an increase in antioxidant capacity as determined by an overexpression of antioxidant enzymes.

### 3.3. Metabolomic Profile after Red Wine Consumption Suggest Better Cardiometabolic Health

**Figure 2:**
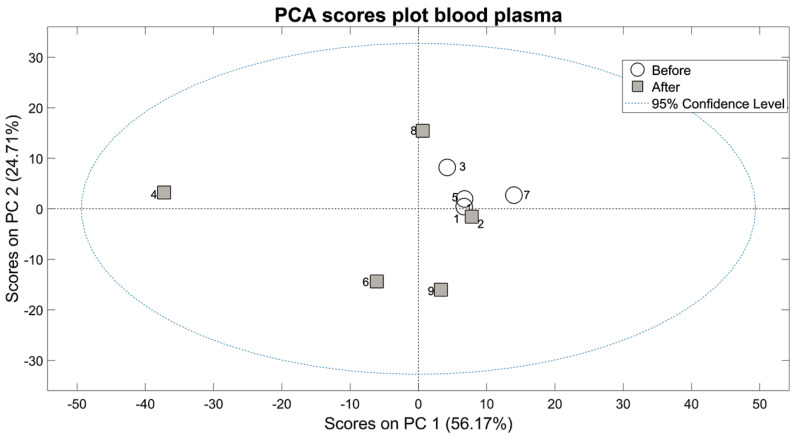
Scores plot for the PCA of NMR spectra of blood plasma. PC1 and PC2 account together for 81% of the total variance of the metabolic profiles of all samples. Each point in the plot represents the global metabolic profile of one sample. Proximity in the PCA scores plot bidimensional space represents similarity in the global metabolic profile. Samples before (white circles) and after (gray squares) wine intervention show clear separation.

We performed a plasma metabolomic analysis of nuns before and after wine consumption. Principal component analysis scores plot (Figure 2) shows a clear separation of samples before and after wine consumption. Metabolomic profile of sample 2, taken after planned wine intervention, resembles those taken before wine intervention. This sample belongs to a nun that refused taking wine and as expected, did not show wine consumption effect regardless of when the sample was taken. Remarkably the gene expression changes that take place in all the rest of nuns, did not occur in this nun.

**Figure 3:**
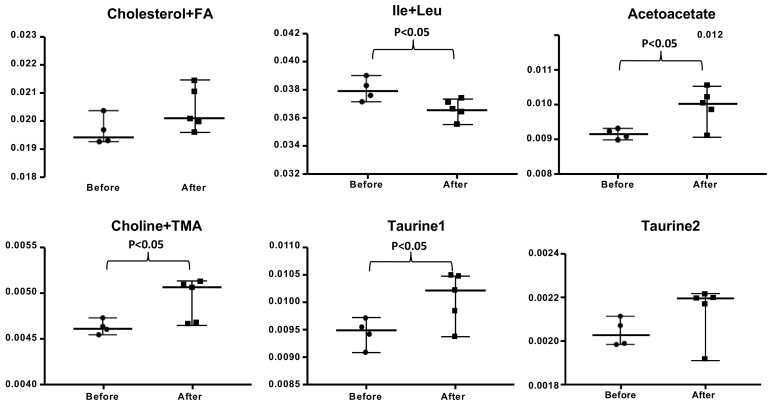
Metabolic relative content (mean relative spectral intensity) for spectral regions enriched in fatty acids (FA) and cholesterol, isoleucine and leucine, acetoacetate, choline and trimethylamine, and taurine. Data are expressed as median and range with 95% CI. The statistical significance is expressed as *p* < 0.05.

Several metabolic spectral regions show significant changes after wine consumption. Fatty acids, cholesterol, branched-chain amino acids (isoleucine and leucine), ketone bodies (acetoacetate), bacterial co-metabolites (choline-trimethylamine), and cellular antioxidants (taurine) contributed to these metabolic spectral regions (see Figure 3). A summary of all the metabolites determined is shown in Table S3.

Therefore, moderate wine consumption in a controlled population of individuals promotes a general better metabolic profile that may help human wellbeing in ageing.

### 3.4. Lack of Adverse Effects of Moderate Red Wine Consumption on General Biochemical and Clinical Parameters

Our nuns drunk two glasses of wine for a period of two weeks. This was deemed sufficient to provoke the changes in gene expression but, as expected, did not change any major biochemical or clinical parameters that we studied. Table S4 shows that blood levels of cholesterol, triglycerides, glucose, urea, creatinine, iron and potassium, cell count, and plasma activity of transaminases (alanine amino transferase and aspartate amino transferase) were not affected by moderate wine consumption for two weeks. 

Therefore, we conclude that changes in the expression of longevity-associated genes are not concomitant with changes in general health of persons as determined by standard clinical chemistry tests.

Clinical examination by a qualified medical doctor (JV) did not show any significant change in the wellbeing or standard clinical parameters of the nuns, leading to the expected conclusion that short-term consumption does not result in any observable negative clinical effects in the nuns. See Table S4.

### 3.5. Wine Consumption Increases Life Span and Upregulates Longevity-Related Gene Expression in Controlled Populations of Drosophila melanogaster

**Figure 4:**
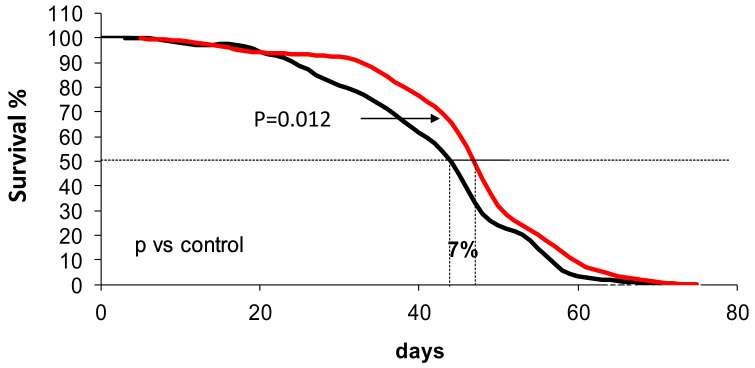
Survival curve of flies treated with or without red wine. Approximately 300 male flies from each group were used in the experiments. The red line corresponds to red wine treated group and black to the control group. The statistics were performed with the Kaplan–Meier test and the significance is expressed as *p* < 0.05 compared to the control.

For obvious reasons, longevity intervention studies cannot be performed in humans. Thus, we turned to *Drosophila melanogaster*, a well-established animal model for aging studies. Figure 4 shows the longevity curve of two different populations of *D. melanogaster* with an average of 300 flies in each population. One, i.e., control, was composed of flies living in the ordinary conditions of our Drosophila center, and the second, was composed of flies that were living in exactly the same conditions as controls except that red wine (10% *v/v*) was added to their diet. The difference between the two curves shows a significant (*p* = 0.012) increase in average lifespan of 7%. Maximal lifespan was not affected. 

**Figure 5:**
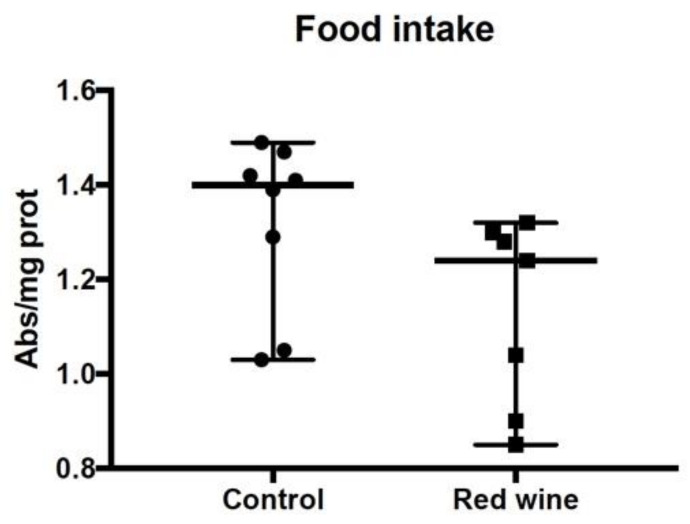
Food intake of flies treated with or without red wine. The absorbance was measured at 625 nanometers (corresponding to the absorption maximum for erioglautia blue). Results are shown as median and range with 95% CI. The differences between the control and the red wine treated groups were not statistically significant (*n* = 7–8).

As red wine consumption could alter the quantity of food ingestion in flies, we tested whether addition of wine resulted in a lowering amount of food ingested by the flies (i.e., equivalent to dietary restriction). To this end, we added erioglaucine blue, i.e., a dye that does not affect the eating behavior of the flies but results in a coloring of the whole animal. Spectrophotometric determination of the amount of dye in the fly’s extracts showed that treatment with wine does not affect eating behavior (Figure 5). 

Thus, the effect wine addition on longevity cannot be attributed to dietary restriction. 

**Figure 6:**
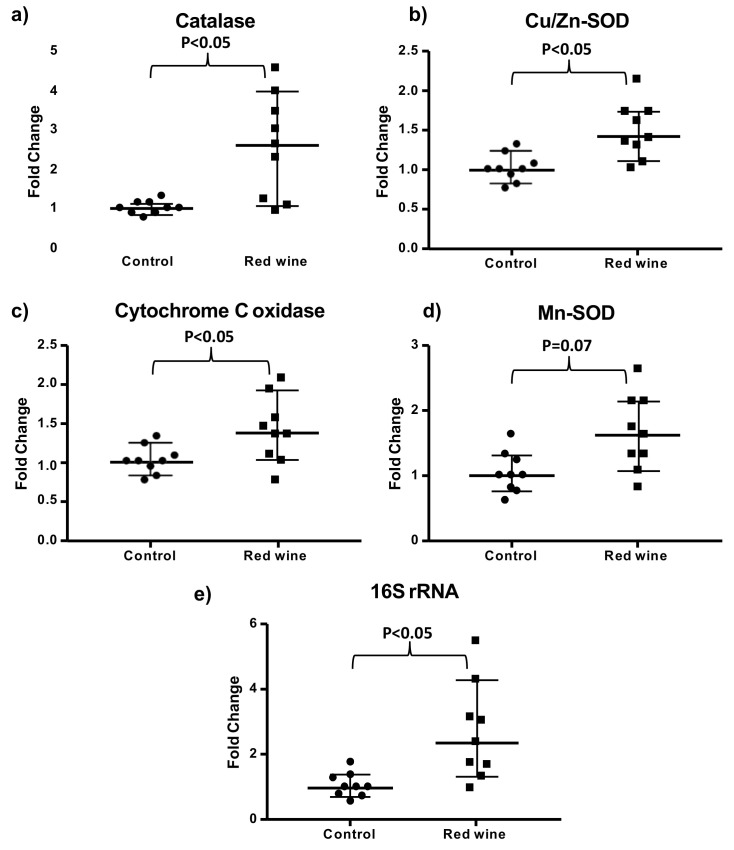
Gene expression in flies treated with or without red wine. Results are shown as median and range with 95% CI for (**a**) catalase, (**b**) Cu/Zn superoxide dismutase, (**c**) cytochrome c oxidase, (**d**) Mn-superoxide dismutase, and (**e**) 16S rRNA. Significance is shown in the graphs.

In order to test if the upregulation of longevity-related gene expression by red wine in humans was also present in flies, we determined the expression of some antioxidant enzymes and a biomarker of aging in control and red wine treated Drosophila for 14 days. Figure 6 shows that we found an up-regulation of catalase, Cu/Zn supersoxide dismutase and cytochrome c oxidase gene expression in red wine treated flies. We did not find significant differences in Mn-superoxide dismutase expression. Moreover, we found that red wine upregulated the expression of 16S rRNA, which has been shown to decrease with aging [23,24] and with oxidative stress [25].

## 4. Discussion

### 4.1. The J-Shaped Curve of the Effects of Wine Consumption on Health

The fact that relatively high doses of wine or any other alcoholic beverage are detrimental for health are obvious and have been well described. However, since the description in 1992 [3] of the French paradox the beneficial effects of relatively small amounts of red wine consumption in some individuals became interesting. The essence of the French paradox is that the French population, who eat a relatively large amount of saturated fats, have a significantly lower incidence of cardiovascular events than neighboring populations who have a similar diet. The advantage of the French diet was traced to the consumption of red wine. 

There is a controversy of the dose of red wine that is advisable for general consumption. Recent evidence indicates that the optimal amount of red wine [26] may range from 1 to 2 glasses of red wine per day. Certainly, higher doses will lead to detrimental effects and lower doses are likely to also cause less advantageous effects than drinking one or two glasses of red wine per day [27]. This is the typical behavior followed by substances that act through their hormetic effect, i.e., the J shape curve, where small concentrations may have beneficial health effects when compared with no consumption or higher concentrations [28,29].

Indeed, our study shows that wine at the dose that we have used, i.e., two glasses a day for a period of two weeks, did not cause any toxic effects determined by clinical inspection of the patients as well as by general clinical biochemistry in their blood. So at least in our study, we can rule out any toxic effects of the consumption of two glasses of wine per day for the period of time studied. It is important to underpin that the catholic nuns enrolled in this study had not drunk red wine in the previous two years.

### 4.2. The Effect of Red Wine in Controlled Populations

The description of the French paradox was an epidemiological study, not an intervention study. Importantly, to understand possible mechanisms by which red wine may show beneficial effects, intervention studies should be performed where the population that receives the wine is strictly controlled. This has been recently done using experimental animals [30] which are, of course, kept under well controlled conditions. 

We have found that longevity-related gene expression is changed in nuns after moderate red wine consumption (Figure 1). A previous study by Di Renzo et al. showed similar results. They found that intake of red wine by healthy people with different diets decreases oxidized LDL level, increases longevity-related genes such as catalase, glutathione peroxidase and Sirt1 and decreases inflammatory CCL5 gene expression [31] 

Regarding the metabolic health, in general, the changes observed reflect better metabolic health and are indicative of enhanced metabolic wellness (see Figure 2 and Figure 3) [32,33]. Free fatty acid and cholesterol are not significantly changed when we compare their levels in nuns before and after wine consumption, confirming the findings of the clinical chemistry analysis (see Table S4). Branched-chain amino acids, especially isoleucine and leucine, were significantly lower after wine consumption than before. A lowering in branched-chain amino acids indicates that protein synthesis is increased which may be interesting in the prevention of sarcopenia [34]. Acetoacetate, a major ketone body found in plasma, is increased after wine consumption. The beneficial effects of relatively high levels of ketone bodies have recently been documented [35]. Therefore, the increase in acetoacetate may again be an index of better metabolic health. Choline is also increased significantly after wine consumption. It was suggested by Zeisel et al. [36] that choline is an essential nutrient for public health. These authors reviewed evidence suggesting that choline has such a wide range of clinical functions in humans that it may be a nutrient required to increase public health of human populations. Finally, taurine also shows some increase after wine consumption but probably due to the low number of nuns that could be recruited, the results are not significant in Taurine 3 peak. Taurine has beneficial effects on cardiovascular disease [37] and has been shown to be an antioxidant amino acid (Figure 3).

One could easily foresee that favorable changes caused by red wine, could be due to other lifestyle changes. For instance, some populations, i.e., the French, could be better at controlling the stress or other general lifestyle modes than neighboring countries, and the beneficial effects could be due to the control of stress or for instance, the amount of physical exercise. Of course, it is difficult to find human populations that can be kept under similar nutritional, social, and personal lifestyles. Nuns, in our case catholic nuns, lend themselves as a unique opportunity to study nutritional interventions in controlled situations. Longevity cannot be studied in humans and therefore we have to resort to experimental animals. *Drosophila melanogaster* is one of the most classical models for longevity studies. The fact that we have observed changes in gene expression in *Drosophila* that are caused by inclusion of wine in the diet that are similar to those observed in nuns, indicates that the beneficial effects of the wine ingestion may be maintained across the animal kingdom, because in one case we studied an invertebrate and in the other we studied human beings. Our studies in *Drosophila* show that red wine ingestion with the diet significantly increases average lifespan (Figure 4). This is to our knowledge the first time that it is shown that wine indeed prolongs life span in a controlled population of animals. Changes in their gene expression (Figure 6) is quite similar to those of humans with, of course, some minor differences, but the general trend can be said to be the same, i.e., moderate red wine consumption increases the expression of longevity-associated genes and that, (in *Drosophila*) leads to an increase in longevity. Indeed, previous work has shown that compounds present in red wine, such as resveratrol or procyanidines increase the expression of antioxidant enzymes, such as catalase and superoxide dismutase [38,39]. Ethanol is also able to increase the expression of sirtuins and p53 in murine fibroblasts [40]. Thus, red wine ingestion may affect gene expression. The mechanism by which red wine modulates longevity-related gene expression remains unclear, however some hypothesis can be raised. On one hand, resveratrol is a phytoestrogen, and we showed in the past, that phytoestrogens from soya upregulate antioxidant gene expression by a mechanism involving oestrogen receptors and MAPK/NFκB signaling pathways [41]. On the other hand, red wine contains a moderate quantity of ethanol, which we also showed to be able to upregulate longevity-related gene expression such as sirtuins, so, maybe it could be also an hormetic effect of red wine, as stated before, mediated by the presence of ethanol [40]. Moreover, ethanol may enhance the effect of red wine by improving extraction and absorption of red wine components [42]. More studies need to be done to clarify the mechanisms by which red wine modulates gene expression. 

It is worthwhile pointing out that in our *Drosophila* experiments we used red wine and also non-alcoholic red wine. The results were favorable in both cases, but in *Drosophila* at least, non-alcoholic wine was better than ordinary red wine in terms of lifespan promotion. We did not use dealcoholized red wine in human studies because of the very low palatability. Our nuns refused to take the non-alcoholic red wine. The fact that ordinary wine is good for your health but that it is less good than dealcoholized wine may point to the damaging effect of alcohol itself on the general health of individuals. 

### 4.3. Should We Recommend the Consumption of Small Amounts of Wine to the General Population?

This is an important point that is derived from many studies on this topic. Our opinion is that the consumption of red wine cannot be a general recommendation for the population, even though epidemiological studies conducted at Cambridge University (UK) indicate that the consumption of two glasses of wine, along with other lifestyle changes, like moderate exercise, taking fruit, and not smoking, increases the life expectancy by 14 years [43,44]. However, if nutrition specialists and other healthcare practitioners were to recommend red wine, it should be after careful medical examinations. Many diseases may be silent or unknown to the patient for instance chronic hepatitis, chronic pancreatitis, or others, and for those individuals, the consumption of red wine should not be encouraged at all. Therefore, even if we observe favorable effects, for the controlled human population, of red wine consumption, the general recommendation should be very careful and should be always proposed in personalized, individual cases, and not made as a recommendation for the general public.

### 4.4. Limitations of the Study

Although we have performed this study in a very well controlled human population, i.e., individuals that live under similar nutritional, social and personal lifestyles, the number of nuns that accepted to participate in the study is not very high. Other studies increasing the number of participants would perhaps give more significant results. Moreover, the nuns were the own controls of the wine consumption, and no white wine or dealcoholized wine was employed in the study as reference ones. 

## 5. Conclusions

The major conclusions of our study are that moderate wine consumption increases the expression of key longevity-associated genes like p53, sirtuin1, catalase, and superoxide dismutase in humans. It also significantly increases longevity-associated gene expression and longevity itself in flies, thus showing that the effects are maintained across the animal kingdom. No serious unwanted side effects were observed. 

## Data Availability

This study does not include datasets publicly archived.

## Supplementary Materials

The following are available online at https://www.mdpi.com/2076-3921/10/2/301/s1. Table S1, Longevity-associated genes whose expression is not affected by moderate wine consumption. Table S2, Lack of effect of moderate red wine consumption on vitamin E, lipid peroxidation, or biomarkers of inflammation, Table S3, General biochemical and clinical parameters. Table S4, Relative contribution of selected metabolic spectral clusters to overall metabolic content.

## References

[1] Jouanna J. (2012). Greek Medicine from Hippocrates to Galen: Selected Papers.

[2] Patay R., Danon G. (1962). [On some biological changes produced, in the swine, by the daily consumption of red wine]. Bull. Acad. Natl. Med..

[3] Renaud S., de Lorgeril M. (1992). Wine, Alcohol, Platelets, and the French Paradox for Coronary Heart Disease. Lancet.

[4] Richard J.L. (1987). [Coronary risk factors. The French paradox]. Arch. Mal. Coeur. Vaiss..

[5] Lavy A., Fuhrman B., Markel A., Dankner G., Ben-Amotz A., Presser D., Aviram M. (1994). Effect of Dietary Supplementation of Red or White Wine on Human Blood Chemistry, Hematology and Coagulation: Favorable Effect of Red Wine on Plasma High-Density Lipoprotein. Ann. Nutr. Metab..

[6] Huang S., Li J., Shearer G.C., Lichtenstein A.H., Zheng X., Wu Y., Jin C., Wu S., Gao X. (2017). Longitudinal Study of Alcohol Consumption and HDL Concentrations: A Community-Based Study. Am. J. Clin. Nutr..

[7] Hayek T., Fuhrman B., Vaya J., Rosenblat M., Belinky P., Coleman R., Elis A., Aviram M. (1997). Reduced Progression of Atherosclerosis in Apolipoprotein E-Deficient Mice Following Consumption of Red Wine, or Its Polyphenols Quercetin or Catechin, Is Associated with Reduced Susceptibility of LDL to Oxidation and Aggregation. Arterioscler. Thromb. Vasc. Biol..

[8] Aviram M., Hayek T., Fuhrman B. (1997). Red Wine Consumption Inhibits LDL Oxidation and Aggregation in Humans and in Atherosclerotic Mice. Biofactors.

[9] Pinotti F., Wikramaratna P.S., Obolski U., Paton R.S., Damineli D.S.C., Alcantara L.C.J., Giovanetti M., Gupta S., Lourenço J. (2021). Potential Impact of Individual Exposure Histories to Endemic Human Coronaviruses on Age-Dependent Severity of COVID-19. BMC Med..

[10] Chen W.-M., Shaw L.-H., Chang P.-J., Tung S.-Y., Chang T.-S., Shen C.-H., Hsieh Y.-Y., Wei K.-L. (2016). Hepatoprotective Effect of Resveratrol against Ethanol-Induced Oxidative Stress through Induction of Superoxide Dismutase in Vivo and in Vitro. Exp. Ther. Med..

[11] Gorelik S., Ligumsky M., Kohen R., Kanner J. (2008). A Novel Function of Red Wine Polyphenols in Humans: Prevention of Absorption of Cytotoxic Lipid Peroxidation Products. FASEB J..

[12] Magyar K., Halmosi R., Palfi A., Feher G., Czopf L., Fulop A., Battyany I., Sumegi B., Toth K., Szabados E. (2012). Cardioprotection by Resveratrol: A Human Clinical Trial in Patients with Stable Coronary Artery Disease. Clin. Hemorheol. Microcirc..

[13] Semba R.D., Ferrucci L., Bartali B., Urpí-Sarda M., Zamora-Ros R., Sun K., Cherubini A., Bandinelli S., Andres-Lacueva C. (2014). Resveratrol Levels and All-Cause Mortality in Older Community-Dwelling Adults. JAMA Intern. Med..

[14] Tresserra-Rimbau A., Medina-Remón A., Lamuela-Raventós R.M., Bulló M., Salas-Salvadó J., Corella D., Fitó M., Gea A., Gómez-Gracia E., Lapetra J. (2015). Moderate Red Wine Consumption Is Associated with a Lower Prevalence of the Metabolic Syndrome in the PREDIMED Population. Br. J. Nutr..

[15] Weaver S.R., Rendeiro C., McGettrick H.M., Philp A., Lucas S.J.E. (2020). Fine Wine or Sour Grapes? A Systematic Review and Meta-Analysis of the Impact of Red Wine Polyphenols on Vascular Health. Eur. J. Nutr..

[16] Vandesompele J., De Preter K., Pattyn F., Poppe B., Van Roy N., De Paepe A., Speleman F. (2002). Accurate Normalization of Real-Time Quantitative RT-PCR Data by Geometric Averaging of Multiple Internal Control Genes. Genome Biol..

[17] Gentleman R.C., Carey V.J., Bates D.M., Bolstad B., Dettling M., Dudoit S., Ellis B., Gautier L., Ge Y., Gentry J. (2004). Bioconductor: Open Software Development for Computational Biology and Bioinformatics. Genome Biol..

[18] Podda M., Rallis M., Traber M.G., Packer L., Maibach H.I. (1996). Kinetic Study of Cutaneous and Subcutaneous Distribution Following Topical Application of [7,8-14C]Rac-Alpha-Lipoic Acid onto Hairless Mice. Biochem. Pharmacol..

[19] Taylor A.W., Bruno R.S., Traber M.G. (2008). Women and Smokers Have Elevated Urinary F(2)-Isoprostane Metabolites: A Novel Extraction and LC-MS Methodology. Lipids.

[20] Leonard S.W., Traber M.G. (2006). Measurement of the Vitamin E Metabolites, Carboxyethyl Hydroxychromans (CEHCs), in Biological Samples. Curr. Protoc. Toxicol..

[21] Hervey G.R. (1953). Determination of Creatinine by the Jaffé Reaction. Nature.

[22] Orr W.C., Mockett R.J., Benes J.J., Sohal R.S. (2003). Effects of Overexpression of Copper-Zinc and Manganese Superoxide Dismutases, Catalase, and Thioredoxin Reductase Genes on Longevity in *Drosophila melanogaster*. J. Biol. Chem..

[23] Borrás C., Sastre J., García-Sala D., Lloret A., Pallardó F.V., Viña J. (2003). Mitochondria from Females Exhibit Higher Antioxidant Gene Expression and Lower Oxidative Damage than Males. Free Radic. Biol. Med..

[24] Calleja M., Peña P., Ugalde C., Ferreiro C., Marco R., Garesse R. (1993). Mitochondrial DNA Remains Intact during Drosophila Aging, but the Levels of Mitochondrial Transcripts Are Significantly Reduced. J. Biol. Chem..

[25] Crawford D.R., Wang Y., Schools G.P., Kochheiser J., Davies K.J. (1997). Down-Regulation of Mammalian Mitochondrial RNAs during Oxidative Stress. Free Radic. Biol. Med..

[26] Kloner R.A., Rezkalla S.H. (2007). To Drink or Not to Drink? That Is the Question. Circulation.

[27] Wood A.M., Kaptoge S., Butterworth A.S., Willeit P., Warnakula S., Bolton T., Paige E., Paul D.S., Sweeting M., Burgess S. (2018). Risk Thresholds for Alcohol Consumption: Combined Analysis of Individual-Participant Data for 599 912 Current Drinkers in 83 Prospective Studies. Lancet.

[28] Calabrese V., Cornelius C., Dinkova-Kostova A.T., Calabrese E.J., Mattson M.P. (2010). Cellular Stress Responses, the Hormesis Paradigm, and Vitagenes: Novel Targets for Therapeutic Intervention in Neurodegenerative Disorders. Antioxid. Redox Signal..

[29] Siracusa R., Scuto M., Fusco R., Trovato A., Ontario M.L., Crea R., Di Paola R., Cuzzocrea S., Calabrese V. (2020). Anti-Inflammatory and Anti-Oxidant Activity of Hidrox^®^ in Rotenone-Induced Parkinson’s Disease in Mice. Antioxidants.

[30] Kołota A., Głąbska D., Oczkowski M., Gromadzka-Ostrowska J. (2020). Analysis of Association between Intake of Red Wine Polyphenols and Oxidative Stress Parameters in the Liver of Growing Male Rats. Appl. Sci..

[31] Di Renzo L., Carraro A., Valente R., Iacopino L., Colica C., De Lorenzo A. Intake of Red Wine in Different Meals Modulates Oxidized LDL Level, Oxidative and Inflammatory Gene Expression in Healthy People: A Randomized Crossover Trial. https://www.hindawi.com/journals/omcl/2014/681318/.

[32] Ahola-Olli A.V., Mustelin L., Kalimeri M., Kettunen J., Jokelainen J., Auvinen J., Puukka K., Havulinna A.S., Lehtimäki T., Kähönen M. (2019). Circulating Metabolites and the Risk of Type 2 Diabetes: A Prospective Study of 11,896 Young Adults from Four Finnish Cohorts. Diabetologia.

[33] Zhang Z.-Y., Monleon D., Verhamme P., Staessen J.A. (2018). Branched-Chain Amino Acids as Critical Switches in Health and Disease. Hypertension.

[34] Gonzalez-Freire M., Adelnia F., Moaddel R., Ferrucci L. (2018). Searching for a Mitochondrial Root to the Decline in Muscle Function with Ageing. J. Cachexia Sarcopenia Muscle.

[35] Puchalska P., Crawford P.A. (2017). Multi-Dimensional Roles of Ketone Bodies in Fuel Metabolism, Signaling, and Therapeutics. Cell Metab..

[36] Zeisel S.H., da Costa K.-A. (2009). Choline: An Essential Nutrient for Public Health. Nutr. Rev..

[37] Xu Y.-J., Arneja A.S., Tappia P.S., Dhalla N.S. (2008). The Potential Health Benefits of Taurine in Cardiovascular Disease. Exp. Clin. Cardiol..

[38] Li Y., Cao Z., Zhu H. (2006). Upregulation of Endogenous Antioxidants and Phase 2 Enzymes by the Red Wine Polyphenol, Resveratrol in Cultured Aortic Smooth Muscle Cells Leads to Cytoprotection against Oxidative and Electrophilic Stress. Pharmacol. Res..

[39] Puiggros F., Llópiz N., Ardévol A., Bladé C., Arola L., Salvadó M.J. (2005). Grape Seed Procyanidins Prevent Oxidative Injury by Modulating the Expression of Antioxidant Enzyme Systems. J. Agric. Food Chem..

[40] Gambini J., Gomez-Cabrera M.C., Borras C., Valles S.L., Lopez-Grueso R., Martinez-Bello V.E., Herranz D., Pallardo F.V., Tresguerres J.A.F., Serrano M. (2011). Free [NADH]/[NAD(+)] Regulates Sirtuin Expression. Arch. Biochem. Biophys..

[41] Borrás C., Gambini J., Gómez-Cabrera M.C., Sastre J., Pallardó F.V., Mann G.E., Viña J. (2006). Genistein, a Soy Isoflavone, up-Regulates Expression of Antioxidant Genes: Involvement of Estrogen Receptors, ERK1/2, and NFkappaB. FASEB J..

[42] Yang X., Zhao Y. (2012). Absorption and Metabolism of Red Wine Polyphenols and Their Potential Health Benefits in Cardiovascular Function. Am. J. Clin. Nutr..

[43] Li Y., Pan A., Wang D.D., Liu X., Dhana K., Franco O.H., Kaptoge S., Di Angelantonio E., Stampfer M., Willett W.C. (2018). Impact of Healthy Lifestyle Factors on Life Expectancies in the US Population. Circulation.

[44] Loef M., Walach H. (2012). The Combined Effects of Healthy Lifestyle Behaviors on All Cause Mortality: A Systematic Review and Meta-Analysis. Prev. Med..

