# Supplementary material for: Moderate Red Wine Consumption Increases the Expression of Longevity-Associated Genes in Controlled Human Populations and Extends Lifespan in Drosophila melanogaster
Source: Antioxidants (Basel). 2021 Feb 16;10(2):301. doi: 10.3390/antiox10020301 (PMC7920262; doi:10.3390/antiox10020301)
Supplement: Supplementary file 1 [file antioxidants-10-00301-s001.pdf]

antioxidants

MDPI

Supplementary Materials: Moderate Red Wine Consumption Increases the Expression of Longevity-associated Genes in Controlled Human Populations and Extends Lifespan in *Drosophila melanogaster*

Juan Gambini [1], Lucia Gimeno-Mallench [1], Gloria Olaso-Gonzalez [1], Angela Mastaloudis [2], Maret G. Traber [2], Daniel Monleón [3], Consuelo Borrás [1,*] and Jose Viña [1]

[1] Freshage Research Group, Department of Physiology, Faculty of Medicine, University of Valencia, CIBERFES-ISCIII, INCLIVA, E46010 Valencia, Spain; juan.gambini@uv.es (J.G.); lucia.gimeno@uv.es (L.G.-M.); gloria.olaso@uv.es (G.O.-G.); jose.vina@uv.es (J.V.)

[2] Linus Pauling Institute, Oregon State University, Corvallis, OR 97331-6512, USA; mastaloudis@gmail.com (A.M.); maret.traber@oregonstate.edu (M.G.T.)

[3] Department of Pathology, Faculty of Medicine, University of Valencia, CIBERFES-ISCIII, INCLIVA, E46010 Valencia, Spain; daniel.monleon@uv.es

* Correspondence: consuelo.borras@uv.es; Tel.: +34 963864646; Fax: +34963864642

Table S1. Longevity-associated genes whose expression is not affected by moderate wine consumption. Data are expressed as concentration means ± SD (Molar*10^{-5}) of 9 different people.

	Before	After	Δ%	*p*
**AKT1**	24 ± 10	27 ± 13	115 ± 24	0.288
**CDKN2A**	0.19 ± 0.13	0.23 ± 0.09	170 ± 99	0.202
**FOXO3**	6.4 ± 4.0	4.9 ± 3.2	86 ± 51	0.184
**FRAP1**	4.0 ± 2.1	3.0 ± 0.9	89 ± 41	0.088
**IRS1**	0.043 ± 0.046	0.085 ± 0.80	273 ± 257	0.105
**SESN2**	3.4 ± 2.3	3.9 ± 2.5	135 ± 61	0.346
**TERF1**	0.05 ± 0.03	0.05 ± 0.04	164 ± 139	0.426
**TERF2**	8.0 ± 4.6	7.9 ± 4.21	112 ± 68	0.487
**TERT**	3.9 ± 7.5	1.9 ± 1.2	232 ± 296	0.237

Table S2. Lack of effect of moderate red wine consumption on vitamin E. lipid peroxidation or biomarkers of inflammation. Data are expressed as means ± SD n=4 before and after wine consumption. Urinary markers are expressed per g creatinine which was not different between the two treatment periods.

	Before	After	*p*
**Plasma**			
**Gamma-Tocopherol (µM)**	0.93 ± 0.14	0.84 ± 0.18	0.228
**Alpha-Tocopherol (µM)**	17.9 ± 2.7	17.1 ± 4.0	0.476
**Urine**			
**Gamma-CEHC (µmol/g Creat)**	0.73 ± 0.10	0.54 ± 0.20	0.21
**Alfa-CEHC (µmol/g Creat)**	2.37 ± 0.67	2.30 ± 0.40	0.86
**PGF2a related (µg/g creat.)**	2.20 ± 0.44	2.58 ± 0.852	0.995
**2.3-dinor-F1 metabolites (µg/g creat.)**	6.2 ± 2.2	17.8 ± 11.4	0.156
**2.3-dinor-F2 metabolites (µg/g creat.)**	55.4 ± 17.3	63.3 ± 25.7	0.853

Table S3. Relative contribution of selected metabolic spectral clusters to overall metabolic content. The value corresponds to the ratio between selected spectral region (labeled as most probable metabolite assignment) and total spectral aliphatic area. The ratio has been multiplied by 1000 for easier readability.

Spectral region metabolite assignment	Before intervention		After intervention		P-value
	Mean	Std	Mean	Std	
2-hydroxyvalerate	8.11	0.49	9.76	2.57	0.301
2-hydroxyvalerate + FFAA ßCH2	19.42	0.50	20.47	1.20	0.192
acetoacetate	9.14	0.12	9.95	0.49	0.025
alanine	4.05	0.22	4.81	1.10	0.272
alloisoleucine + 2-hydroxyvalerate	9.99	0.31	9.88	0.33	0.674
cholesterol	46.57	2.91	44.20	3.02	0.329
cholesterol + FFAA (-CH3)	19.60	0.44	20.42	0.70	0.113
choline + TMA	4.62	0.07	4.92	0.21	0.049
citrate	2.49	0.11	8.86	5.43	0.078
creatine + glucose	5.51	0.36	6.90	2.01	0.270
creatinine	3.59	0.18	4.38	1.27	0.311
cysteine	8.93	0.53	10.22	2.10	0.326
cysteine + lysine	3.63	0.11	3.63	0.17	0.982
cysteine + creatinine	4.92	0.23	5.15	0.47	0.451
FFAA (=CH-CH2-CH=)	17.68	0.48	19.88	3.85	0.351
FFAA (=CH-CH2-CH=) + malate	0.32	0.01	0.70	0.38	0.127
FFAA (=CH-CH2-CH2-)	2.70	0.06	2.77	0.15	0.452
FFAA (=CH-CH2-CH2-) + isoleucine	6.27	0.12	6.72	0.53	0.183
FFAA (-CH2)n	28.01	0.17	26.67	1.01	0.054
FFAA (-CH2)n + isoleucine	82.28	3.50	84.98	4.89	0.438
FFAA (-CH3)	51.11	1.64	49.17	2.39	0.261
FFAA (-CH3) + valerate	56.82	1.06	58.37	1.42	0.154
FFAA ßCH2	9.26	0.15	9.56	0.29	0.150
FFAA αCH2 + valerate	6.36	0.08	6.57	0.26	0.200
glucose + glycine	3.52	0.10	3.87	0.27	0.067
glucose + lysine	3.88	0.20	4.52	0.90	0.258
glucose + TMAO	3.16	0.29	3.65	0.67	0.262
glutamine	11.43	0.42	11.81	0.90	0.515
isobutyrate + glutamine	5.33	0.18	5.71	0.45	0.200
isobutyrate + malate	3.40	0.11	3.57	0.29	0.368
isoleucine + leucine	37.96	0.71	36.55	0.64	0.028
lactato	2.90	0.11	3.30	0.79	0.398
leucine	4.81	0.16	4.84	0.22	0.868
leucine + 2-hydroxyvalerate	22.55	0.76	22.67	1.08	0.867
leucine + alloisoleucine	11.72	0.28	11.30	0.34	0.118
leucine + glucose	1.91	0.05	2.13	0.32	0.261
leucine + lysine	20.80	0.81	20.81	0.98	0.987
lysine	26.47	0.78	26.20	0.92	0.694
malate	8.46	0.80	10.24	2.67	0.294
malate + proline	1.99	0.07	2.10	0.19	0.370
prolina + lactato	11.53	0.38	12.89	2.84	0.431
proline + alloisoleucine	13.54	0.65	13.92	0.97	0.575
proline + glucose	9.44	0.23	10.07	0.42	0.051
taurine1	9.44	0.26	10.07	0.47	0.043
taurine2	2.04	0.05	2.14	0.11	0.183
threonine	11.62	0.94	13.71	3.51	0.342
unknown1	3.53	0.08	3.93	0.60	0.286
valerate	5.45	0.11	5.52	0.28	0.674
valine	20.15	0.60	22.04	3.16	0.332
valine + FFAA αCH2	17.81	0.39	25.14	8.07	0.153

Keys: FFAA, fatty acids; TMA, trimethylamines; TMAO, trimethylamine-oxide.

Table S4. General biochemical and clinical parameters. Data are expressed as means ± SD of 9 different people. The statistical significance is expressed as *P < 0.05 or **P < 0.01 vs. control.

	Before	After	*P*
**Leucocytes**	4.88 ± 0.68	4.83 ± 0.74	0.460
**Red cells**	4.39 ± 0.17	4.25 ± 0.20	0.166
**Hemoglobin**	13.2 ± 0.5	13.1 ± 0.6	0.400
**Hematocrite**	41.1 ± 1.4	40.4 ± 2.1	0.285
**Average cor. vol.**	93.6 ± 1.7	95.0 ± 1.4	0.126
**Average cor. HB**	30.1 ± 0.6	30.9 ± 0.4*	0.039
**Average cor. [HB]**	32.2 ± 0.2	32.5 ± 0.5	0.101
**Wide distribution hematie**	13.7 ± 0.4	14.2 ± 0.5	0.071
**Platelets**	208 ± 56	263 ± 27	0.065
**MPV**	11.2 ± 1.5	11.3 ± 1.5	0.464
**Immature neutrophils**	0.039 ± 0.023	0.048 ± 0.005	0.251
**Segmented neutrophils**	2.60 ± 0.44	2.65 ± 0.54	0.451
**Basophils**	0.015 ± 0.006	0.023 ± 0.001	0.114
**Eosinophils**	0.16 ± 0.06	0.15 ± 0.07	0.400
**Lymphocytes**	1.63 ± 0.30	1.55 ± 0.29	0.350
**Monocytes**	0.43 ± 0.05	0.32 ± 0.20	0.160
**Reticulocytes**	40.3 ± 19.7	50.7 ± 21.0	0.249
**Glucose**	77.8 ± 6.7	77.2 ± 10.5	0.463
**Total cholesterol**	228 ± 15	242 ± 33	0.241
**HDL**	74.7 ± 4.2	88.4 ± 4.8**	0.0026
**LDL**	135 ± 10	136 ± 27	0.468
**Triglicerides**	91.2 ± 57	84.5 ± 37	0.424
**Total proteins**	7.23 ± 0.34	7.23 ± 0.28	0.500
**Alfa 1 glicoprot ac.**	56.8 ± 6.3	95.8 ± 9.1**	0.00020
**PCR**	0.125 ± 0.066	0.143 ± 0.060	0.355
**Insuline**	4.94 ± 2.29	5.87 ± 2.90	0.317
**Albumin**	4.35 ± 0.01	4.20 ± 0.18	0.067
**Afla 1 globulin**	0.222 ± 0.038	0.230 ± 0.029	0.382
**Afla 2 globulin**	0.653 ± 0.099	0.753 ± 0.0112	0.115
**Beta globuline**	0.803 ± 0.118	0.788 ± 0.123	0.433
**Gamma globuline**	0.955 ± 0.570	1.27 ± 0.15	0.167
**Albumine/globuline**	1.53 ± 0.17	1.40 ± 0.17	0.163
